# Reducing the risk of non-sterility of aseptic handling in hospital pharmacies, part B: risk control

**DOI:** 10.1136/ejhpharm-2019-002179

**Published:** 2020-05-08

**Authors:** Frits A Boom, Judith M Ris, Tjitske Veenbaas, Paul P H Le Brun, Daan Touw

**Affiliations:** 1 Zaans Medical Centre, Zaandam, Noord-Holland 1502 DV, The Netherlands; 2 Albert Schweitzer Ziekenhuis, Dordrecht, Zuid-Holland 3318 AT, The Netherlands; 3 Department of Clinical Pharmacy and Toxicology, Leiden University Medical Centre, Leiden, The Netherlands; 4 Clinical Pharmacy and Pharmacology, University Medical Centre Groningen, Groningen, The Netherlands; 5 Pharmacokinetics Targeting and Toxicology, University of Groningen, Groningen, The Netherlands

**Keywords:** aseptic preparation, audit, self-inspection, compounding (individualised preparation), disinfection, good manufacturing practice (GMP), laminar flow technology, manufacturing, small scale, protocols & guidelines, reconstitution, risk management, safety cabinets, validation preparation process

## Abstract

**Objectives:**

To determine prospectively the risk reducing measures of non-sterility during aseptic handling and to develop a method for prioritising these measures.

**Methods:**

In the first part of this series of articles, we identified all sources of risk which could contaminate a product during aseptic handling, and calculated the remaining risks of non-sterility using a risk assessment (RA) model. We concluded that additional research of some risk sources was needed before risk control (RC) could be executed on all risk sources.

The chances of technical problems with a laminar airflow cabinet or safety cabinet (LAF/SC) were collected from 10 hospital pharmacies using a questionnaire. The chances of blocking first air were examined by airflow visualisation (smoke studies). For checking the way of working during aseptic handling, a checklist for an audit was developed.

Risk control was executed by a multidisciplinary team of (hospital) pharmacists and technicians, a consultant experienced in aseptic processing and an independent facilitator. They determined the risk reducing measures for each source of risk and the influence of these measures on the remaining risk (expressed as risk prioritisation number).

**Results:**

The chances of defects of the LAF/SC were low. Airflow visualisation is a sensible method to find the correct location of materials and equipment inside the LAF/SC and to detect a way of working without blocking first air on critical spots. Audits will provide valuable information about the way aseptic handling is executed and the remaining risks as a consequence. The risk of non-sterility caused by needle or spike contact with critical spots of vials and ampoules (stopper or ampoule neck), blocking first air under downflow and touching critical spots cannot be eliminated completely.

**Conclusion:**

The RA/RC model shows the impact of risk reducing measures on the probability of non-sterility during aseptic handling. The calculated risk prioritisation numbers are helpful in prioritising these measures. Audits result in risk reduction for nearly all sources of risk.

## Introduction

Many sources of risk can contribute to microbiological contamination of a product during aseptic handling.^1^ It is not clear which of these factors is the most important. The same is true for the effectiveness of measures to reduce these risks. For solving this lack of knowledge, we described in part A of this series of articles a risk assessment (RA) model, founded on Failure Mode and Effect Analysis.^1^ The different risks were discussed by a multidisciplinary team and expressed as risk prioritisation numbers (RPN). An incorrect disinfection technique of non-sterile materials and the chances of touching critical spots were estimated as the greatest risks (a definition of critical spot is given in online supplementary file 1). It was also concluded that there is a lack of information about the risk of defects in the laminar airflow cabinet (LAF) or safety cabinet (SC). Also, more information is needed about the chances of disturbing the unidirectional flow and blocking of first air on critical spots (definitions of unidirectional flow and first air are given in online supplementary file 1).^1^ Both aspects are further examined here (see ‘Qualified air during aseptic handling’).

10.1136/ejhpharm-2019-002179.supp1 Supplementary data

Another shortcoming found by the team during discussions about RA was the lack of information on the way of working during aseptic handling.^1^ Results from microbiological controls, process validation with a broth solution in particular, are informative. However, not all aspects of the way of working can be measured by these controls.^2^ Therefore, the team advised regular auditing of each operator (see ‘Auditing of operators during aseptic handling’).

The main objective of this study was risk control (RC)—additional measures to reduce the risk of non-sterility during aseptic handling to an acceptable level.^2 3^ We developed an RC method for prioritising these measures. This will be described in the final part of this article.

## Materials and methods

### Qualified air during aseptic handling

#### Risk of defects of the LAF or SC

Ten hospital pharmacies were asked to complete a questionnaire about technical problems with their LAF and/or SC over a period of 5 years (the questionnaire is described in online supplementary file 2).

10.1136/ejhpharm-2019-002179.supp2 Supplementary data

#### Airflow visualisation: searching the risk sources of disturbing unidirectional flow and blocking first air on critical spots

For airflow visualisation, a Condensation Fog Generator CFG 290 (Lighthouse Benelux BV) was used. Fog was made out of Safex nebelfluid extra clean F&D (Safex Chemie GMBH). All substances of this fluid are non-toxic and evaporate completely. Air flow patterns were evaluated during the following situations:

1. LAF (crossflow) and SC (downflow) loaded with materials at rest and during aseptic handling (a definition of at rest is given in online supplementary file 1).
2. Filling of syringes with a Baxa repeater pump in LAF (crossflow) and under a downflow plenum.
3. The influence of quick movements of the hands and forearms by the operator inside the LAF/SC on unidirectional flow.
4. The influence of opening and closing the entrance door to the background area (this is the room in which the LAF/SC is housed) and fast moving (fast walking) of operators in the background on unidirectional flow in the preparation area inside the LAF/SC.

Pictures and videos were taken of all situations.

### Audit of operators during aseptic handling

Auditing was executed with the help of a checklist, which covered the whole process of aseptic handling, including activities before and after preparations inside the LAF/SC (see online supplementary file 3). The checklist was developed for two people working together: a primary and a secondary operator (the principle of working with a primary and secondary operator is explained in part A of this series of articles^1^). Activities with a high risk of microbiological contamination of the product were marked as critical.

10.1136/ejhpharm-2019-002179.supp3 Supplementary data

The audit is executed by competent staff, qualified and experienced in aseptic handling. One audit cycle consists of an audit of all operators carrying out aseptic handling. The cycle is concluded with an evaluation of the results.

### Risk control of aseptic handling

The RA was the starting point for the RC.^1^ By using (1) the results from the sections ‘Qualified air during aseptic handling’ and ‘Audit of operators during aseptic handling’ (see below), (2) results from previous articles about aseptic transfer procedures,^4 5^ (3) additional scientific information and (4) experiences of the team members, the multidisciplinary team listed additional measures for risk reduction for each source of risk. New values for occurrence and detection were determined, and the remaining risks were expressed in new RPN.

The goal was risk reduction to an acceptable level, preferable to a safe (green) RPN score (see part A of this series of articles^1^). However, the effort made to reduce the amount of risk should be proportional to the impact of the risk.^2^

## Results

### Qualified air during aseptic handling

#### Risk of defects of the LAF or SC

All 10 hospital pharmacies completed the questionnaire. In the 10 hospital pharmacies, 49 LAF/SC units were in use, of which 7 were LAF and 42 were SC. Mean age was 7 years (range 2–20 years). Thirty-four LAF/SC had regular maintenance and requalification once a year and 15 twice a year. In all cases, maintenance and requalification was executed by companies specialised in the LAF/SC.

For the LAF, during daily work, no defects were observed. During maintenance and requalification, ‘too many particles’ were found on one occasion, caused by damaged seals (over 8 years old) around the HEPA filter.

For the SC, during daily work, a ventilator was defective on one occasion and a flow alarm occurred twice. All defects were shown on the control panel and work inside the SC was stopped immediately and a repair requested. During maintenance and requalification, ‘too much or too little under pressure’ was found three times and ‘air flow velocity too low’ was found on one occasion. The latter was caused by a clogged prefilter.

#### Airflow visualisation: searching the risk sources of disturbing unidirectional flow and blocking first air on critical spots

**Figure 1:**
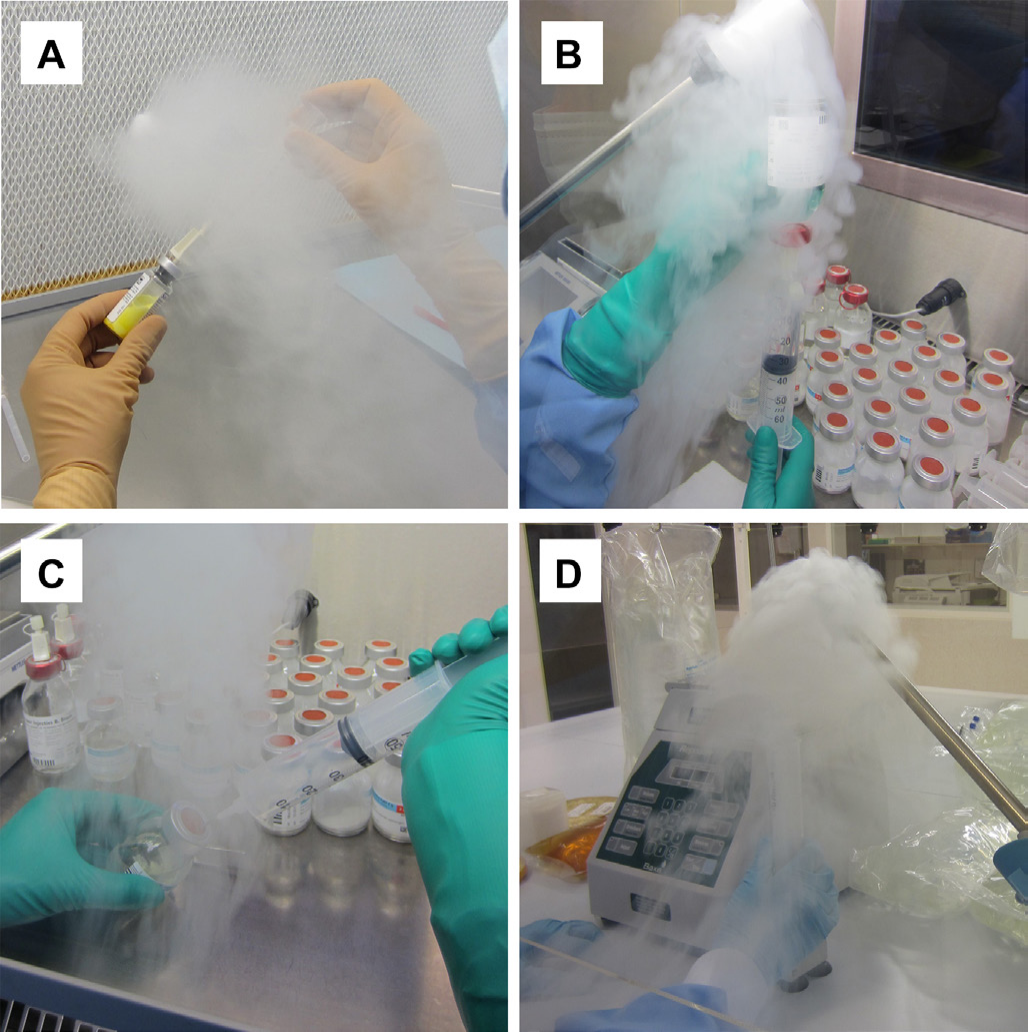
Airflow visualisation during aseptic handling. (A) Airflow in the laminar airflow cabinet: no blocking of first air on critical spots. (B) Airflow in the safety cabinet (SC), syringe and vial in a vertical position: blocking of first air on critical spots. (C) Airflow in the SC, syringe and vial in oblique position: no blocking of first air on critical spots. (D) Airflow under downflow during filling syringes by a Baxa repeater pump: blocking of first air on critical spots. Additional pictures are available in videos on YouTube.^6^

For the LAF (crossflow) at rest, unidirectional flow can be disturbed by materials and equipment, which can result in blocking first air on the work zone and on critical spots (a definition of work zone is given in online supplementary file 1). Therefore, materials should be located on the left and/or right side of the work zone. During aseptic handling, the chances of blocking first air on critical spots by moving parts (materials) and the operator (hands and forearm) were low (see figure 1A).

For the SC (downflow) at rest, materials and equipment do not disturb the unidirectional flow on critical spots. However, during aseptic handling, blocking first air on critical spots by moving parts (materials) and the operator (hands and forearm) can occur (figure 1B). Therefore, syringes and vials should be held in a skewed position (figure 1C).

Filling syringes without blocking first air on critical spots in LAF on the so called ‘syringe filling fixture’ of a Baxa repeater pump is easy to do, but impossible to do in downflow (figure 1D and YouTube video^6^).

Quick movements of the hands and the forearms of the operator inside the LAF/SC influences unidirectional flow. Therefore, if shaking is necessary (eg, dissolving freeze dried products), this should be done away from the work zone inside the LAF/SC. During the experiments with opening and closing the entrance door and fast moving (fast walking) of operators in the background area, we did not observe a visible influence on unidirectional flow inside the LAF/SC.

### Audit of operators during aseptic handling

Thirteen of 50 questions on the checklist were marked as critical (see online supplementary file 3). The criteria for a positive judgement were: all critical activities have to be executed in the correct way (in accordance with the standard operating procedures (SOPs)) and of the remaining questions, a maximum of five may fail. We found that during the first audit cycle, approximately 25% of the critical activities failed. In the following cycles, nearly all operators met the criteria; if not, they received supplementary training.

If the primary and secondary operator change their tasks during the audit, an audit of both can be executed by one auditor. This requires about 3 hours. Evaluations between auditors and auditees immediately afterwards, and finishing the checklist with concluding remarks, will take another hour.

### Risk control of aseptic handling

**Figure 2:**
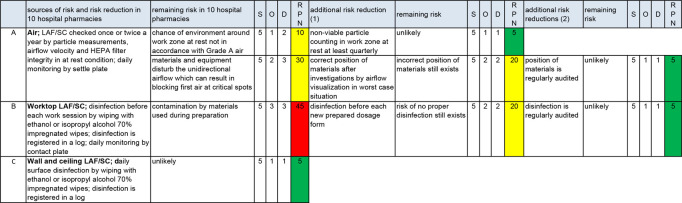
Risk assessment and risk control of the work area. D, detection; LAF, laminar airflow cabinet; O, occurrence; RPN, risk prioritisation number; S, severity; SC, safety cabinet. A, B and C=sources of risk of non-sterility.

**Figure 3:**
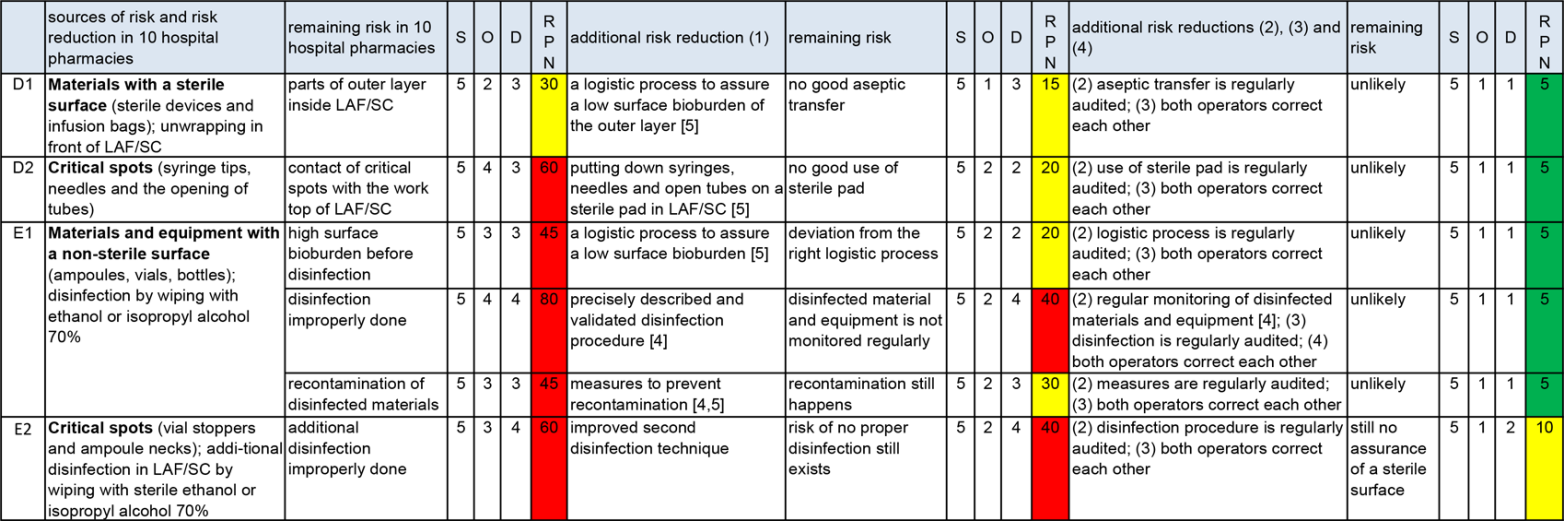
Risk assessment and risk control of the transfer of materials. D, detection; LAF, laminar airflow cabinet; O, occurrence; RPN, risk prioritisation number; S, severity; SC, safety cabinet. D1, D2, E1 and E2=sources of risk of non-sterility.

**Figure 4:**
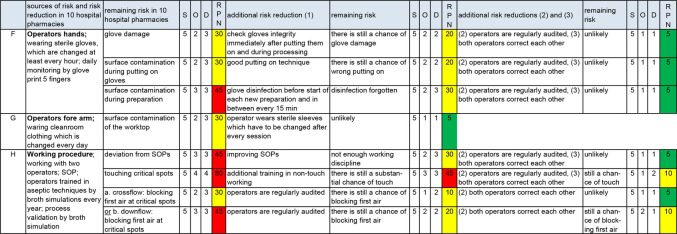
Risk assessment and risk control of the operator. D, detection; O, occurrence; RPN, risk prioritisation number; S, severity; SOP, standard operating procedure. F, G and H=sources of risk of non-sterility.

RC is worked out in three areas: work area (figure 2), transfer of materials (figure 3) and operator (figure 4). The results in the columns up to and including the first RPN are taken from figure 2 in part A of this series of articles^1^).

To reduce the width of figures 3 and 4, we put additional risk reduction (2, 3) and (4) together in one column; figures 3 and 4 with full separation of all risk reducing measures are available as separate files in online supplementary files 4; 5.

10.1136/ejhpharm-2019-002179.supp5 Supplementary data

10.1136/ejhpharm-2019-002179.supp4 Supplementary data

In general, each risk reducing measure will reduce the value for occurrence or detection by 1 point. However, auditing will improve detection, and if points of improvement are followed-up, it will also reduce occurrence. Therefore, if audit cycles are implemented (see ‘Audit of operators during aseptic handling’), the values for occurrence and detection will reduce by 1 point. Background information about other risk reducing measures with more than 1 point reductions is given in the discussion.

## Discussion

### Qualified air during aseptic handling

Aseptic handling is done with closed systems. Therefore, compared with open aseptic processes, the risk of contamination by the airborne route is low. However, this may not lead to less attention for grade A air on critical spots inside the LAF/SC (a definition of grade A air is given in online supplementary file 1).

#### Risk of defects of the LAF or SC

Defects, found in our study, which could influence the quality of the product inside the LAF/SC were too many particles (on one occasion in the LAF) and too low airflow velocity (on one occasion in the SC). Both defects may not have happened if corrective maintenance had been executed according to the advice of the manufacturers of the LAF/SC.

The required frequency of maintenance and requalification of the LAF/SC in hospital pharmacies in the UK and USA is 6 months.^7 8^ In other countries, such as The Netherlands and Germany, the frequency is once a year.^9 10^ The maximum possible period of defects on the LAF/SC will be shorter if 6 month checks are carried out, but the risk of defects in the LAF/SC for some period still exists.

Not all defects have a direct influence on the quality of the product, but it is important to be sure that the environment around the work zone complies with at rest criteria for airborne particles of an EU grade A environment.^11^ Therefore, some guidelines advise particle counting at rest inside the LAF/SC every 3 month.^7 12^ This test appears to be more useful compared with increasing the frequency of a full and expensive requalification of the LAF/SC.

Some LAF/SC are equipped with a fixed probe for continuous particle counting. This probe, however, is located out of the work zone, which makes the results of continuous particle counting inadequate in the case of aseptic handling.

#### Airflow visualisation: searching the risk sources of disturbing unidirectional flow and blocking first air on critical spots

Our findings on airflow visualisation are also applicable to other hospital pharmacies (see figure 1 and videos on YouTube).^6^ In situations where filling or another apparatus is used inside the LAF/SC, it is better to check unidirectional airflow and the chances of blocking first air using the pharmacy’s own experiments.^13^ Because smoke rests inside the LAF/SC, we advise against the use of smoke cartridges for these experiments. Fog, made by a fog generator, leaves no residues if the right fog fluid is used. A fog generator can be rented.

### Audit of operators during aseptic handling

Accurate and up to date SOPs are essential references in judging operators during audits. Frequent deviations should be incorporated into the quality improvement system, to ensure follow-up activities. Further improvement can be done if operators correct each other in a constructive way.^14^

### Risk control of aseptic handling

Audits provide risk reduction for nearly all sources of risk (figures 2–4), making them a powerful risk reducing measure. If correcting each other is general practice, this will also be an effective risk reducing measure (figures 3 and 4). Further background information about figures 2–4 is given below (‘Work area’, ‘Transfer of materials’ and ‘Operator’).

#### Work area

##### Air

Some guidelines require a grade C background area.^10 12^ Experiments with the fog generator showed that the chances of getting viable and non-viable particles from the background inside the LAF/SC were very low. Also, the work zone may not be close to the front of the LAF/SC. Therefore, the influence of the background area on the quality of air in the work zone is negligible. Hence a grade C instead of a grade D background is not listed in figure 2 as an additional risk reducing measure.

The additional risk reduction of the risk source air in figure 2 is in accordance with the advice in ‘Qualified air during aseptic handling’.

##### Worktop LAF/SC

The consequences of the risk of dragging microorganisms inside the LAF/SC by materials can be reduced if the worktop is disinfected by wiping frequently (in part A of this series of articles, we described that wiping is a combination of disinfection and cleaning^1^). This leads to lower occurrence and also lower detection (disinfection is easy to observe).

The disinfectants most widely used are ethanol and isopropyl alcohol; both are not sporicidal. Guidelines advise periodic use of sporicidal disinfectants.^11 15^ However, studies showed that wiping with alcohol impregnated wipes removes bacterial spores mechanically from surfaces spiked with these spores.^16 17^ Our study on the optimal disinfection process of ampoules and vials showed that spore forming bacteria disappear as quickly as other microorganisms after disinfection by alcohol impregnated wipes.^4^ Hence periodic use of a sporicidal disinfectant is not listed in figure 2 as an additional risk reducing measure. If impregnated wipes are too wet, the mechanical effect will be less or even absent. On the other hand, if not completely impregnated, not all surfaces will be disinfected. Therefore, to eliminate the effect of insufficient wetting, the use of commercially available alcohol impregnated wipes is advised.^18^

#### Transfer of materials (figure 3 and online supplementary file 4)

Transfer of materials have the sources of risk D1 (materials with a sterile surface) and E 1 (materials and equipment with a non-sterile surface). Some parts of these materials may come into contact with a sterile fluid (critical spots, D2 and E2).

##### Materials with a sterile surface

The additional risk reduction for materials with a sterile surface, as described in figure 3, are in accordance with the recommendations in a previous article.^5^ The multidisciplinary team considered the application of a sterile pad (see Boom *et al* 5) as an important risk reducing measure (occurrence reduced by 2 points) which can be detected easily (detection reduced by 1 point).

##### Materials with a non-sterile surface

Dragging microorganisms across non-sterile materials (source of risk E1) is a serious risk.^1^ Keeping ampoules and vials in their original boxes as long as possible and handling these materials with gloved hands can mean a low bioburden before disinfection.^5^ These measures are easy to detect and therefore will reduce occurrence and detection.

Improving the effectiveness of the disinfection of non-sterile materials needs a validated procedure and a precise description in an SOP.^4^ The team considered these measures important and reduced the value of occurrence by 2 points. Regular surface monitoring after disinfection is strongly advised.^4 7 11^ Therefore, the team reduced the value of detection by 2 points. Recontamination of disinfected materials can occur by the worktop outside the LAF/SC. Measures to prevent this are described in previous articles.^4 5^

##### Critical spots of materials with a non-sterile surface

Additional disinfection of critical spots (E2) in the LAF/SC cannot be validated by microbiological investigations.^5^ This makes the importance of auditing, and operators who correct each other, even greater. However, the remaining risk of non-sterility caused by needle or spike contact with critical spots of vials and ampoules has to be accepted (figure 3).

#### Operator (figure 4 and online supplementary file 5).

##### Operators’ hands

**Figure 5:**
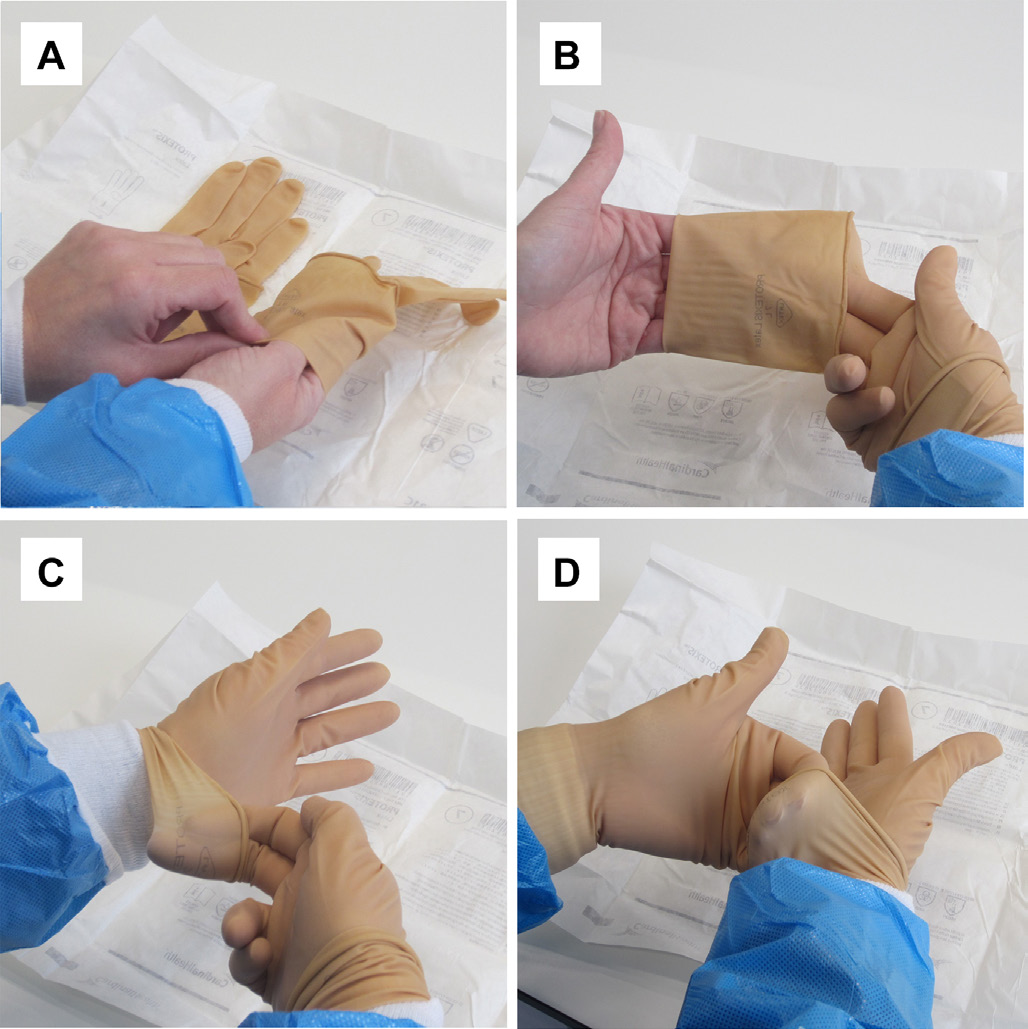
(A–D) Putting on gloves with a low chance of outer surface contamination: steps for putting on the gloves.

Additional risk reduction of gloves is shown in figure 4. Visible damage to gloves can be recognised by checking the gloves after putting them on and during use.^1^ A good technique for putting on gloves is shown in figure 5. To keep the surface bioburden low, glove disinfection at least every 15 min is important.

The risk of blocking first air on critical spots by the hands is discussed below (see ‘Working procedures’).

##### Operators’ forearm

The risk reduction of the operators’ forearm is wearing sterile sleeves or long-sleeved gloves; this reduces occurrence and detection because the visibility of using sleeves/long sleeved gloves is clear (RPN reduces to 5). The risk of blocking first air on critical spots by the forearms is discussed below (see ‘Working procedures’).

##### Working procedures

SOPs can be improved by describing the processes in detailed and unequivocal language. Working according to SOPs requires good working discipline. Not all aspects of aseptic handling can be measured by microbiological controls (eg, touching of critical spots). Additional training of operators, with demonstrated suboptimal non-touch techniques, can be effective (occurrence and detection will be reduced by 1 point each). Observations (audit as well as operators who correct each other) can further reduce the risk but the team concluded that touching critical spots will remain a risk of non-sterility (RPN=10, yellow, nearly safe).

We discussed the risk of blocking first air on critical spots by moving parts (materials) and personnel (hands and forearm) (see ‘Qualified air during aseptic handling’). We concluded that blocking first air under downflow can easily occur and cannot completely be eliminated. The results in figure 4 are in accordance with this conclusion.

### Applying RA and RC in practice

After collecting information on the way aseptic handling is executed in a particular hospital pharmacy, the RA/RC model described in figure 2 and online supplementary files 4 and 5 can be completed and an improvement programme can start on the basis of risk prioritisation. In a future article we will describe this process in approximately 10 hospital pharmacies.

## Conclusion

The RA/RC model shows the impact of risk reducing measures on the probability of non-sterility during aseptic handling. The calculated RPN are helpful in prioritising these measures. Audits resulted in risk reduction for nearly all sources of risk.

**Key messages**

**What is already known on this subject**

- Aseptic handling should be executed with aseptic precautions in a laminar airflow cabinet, safety cabinet or isolator
- The operator is the highest risk source of non-sterility.

**What this study adds**

- A systematic survey to reduce the risks of non-sterility of aseptic handling
- The results can be used in prioritising risk reducing measures
- The risk of non-sterility caused by needle or spike contact with critical spots of vials and ampoules (stopper or ampoule neck), blocking first air under downflow and touch of critical spots cannot be eliminated completely

## Data Availability

Data are available upon reasonable request. An Excel file with the data for figures 2, 3 and 4, and supplementary files 4 and 5, is available.
