# Supplementary material for: Reducing the risk of non-sterility of aseptic handling in hospital pharmacies, part B: risk control
Source: Eur J Hosp Pharm. 2020 May 8;28(6):325–30. doi: 10.1136/ejhpharm-2019-002179 (PMC8552189; doi:10.1136/ejhpharm-2019-002179)
Supplement: Supplementary data [file ejhpharm-2019-002179supp001.pdf]

SUPPLEMENTARY FILE 1

Definitions

- At rest is a room (an environment) complete with all HVAC systems, utilities functioning and with manufacturing equipment installed as specified but without personnel in the facility and the manufacturing equipment is static [1].
- Critical spot is a surface (spot) that may come into contact with a sterile fluid [1]; in the case of aseptic handling a conus of a syringe, a needle, an opening of a tube, an injection puncture, a vial stopper or the neck of an ampoule.
- First air is air from a HEPA filter on a surface without having been obstructed by a non-sterile surface.
- Grade A air is air which is passed through a filter qualified as capable of producing grade A non-viable quality air, but where there is no requirement to continuously perform non-viable monitoring or meet grade A viable monitoring limits [1].
- Unidirectional flow is an airflow moving in a single direction, in a robust and uniform manner, and at sufficient speed, to reproducibly sweep particles away from the critical processing or testing area [1].
- Work zone is that part of the worktop inside LAF/SC where the preparation activities are executed.

Reference

1. EU Good manufacturing practice (GMP) Annex 1 Revision. Manufacture of sterile medicinal products. December 2017. http://academy.gmp-compliance.org/guidemgr/files/2017_12_PC_ANNEX1_CONSULTATION_DOCUMENT.PDF (accessed 3 December 2019).
