# Supplementary material for: Reducing the risk of non-sterility of aseptic handling in hospital pharmacies, part B: risk control
Source: Eur J Hosp Pharm. 2020 May 8;28(6):325–30. doi: 10.1136/ejhpharm-2019-002179 (PMC8552189; doi:10.1136/ejhpharm-2019-002179)
Supplement: Supplementary data [file ejhpharm-2019-002179supp002.pdf]

SUPPLEMENTARY FILE 2

Questionnaire about technical problems with LAF and SC

The following questions were asked:

1. Specifications LAF/SC and the date either of these have been put into operation.
2. Frequency of regular maintenance and physical validation like filter integrity, pressure differential across HEPA filter(s), particle counts, air velocity, air flow pattern and in case of SC potassium iodine test.
3. Continuous or periodical (monthly or 3 monthly) particle counts at rest
   - yes or no
4. Number and kind of deviations in the last 5 years
   - Found during daily work
   - Found during regular maintenance
