# Supplementary material for: Reducing the risk of non-sterility of aseptic handling in hospital pharmacies, part B: risk control
Source: Eur J Hosp Pharm. 2020 May 8;28(6):325–30. doi: 10.1136/ejhpharm-2019-002179 (PMC8552189; doi:10.1136/ejhpharm-2019-002179)
Supplement: Supplementary data [file ejhpharm-2019-002179supp003.pdf]

SUPPLEMENTARY FILE 3

Checklist 'Aseptic handling', which can be used during an audit

	**Operators clothing and hygiene**	critical (C)	judgment
1	Wrist watches, jewellery and cosmetics should not be worn		
2	Wash hands		
3	Disinfect hands		
4	Change clothes; prevent contact between cleanroom clothes and floor		
5	Wear face mask and gloves before entering background area		
6	Disinfect hands during each glove change		
	**Set up**		
7	Check background area		
8	Switch on LAF/SC on working mode		
9	Disinfect by wiping inside LAF/SC		
10	Wipes well impregnated		
11	Register disinfection in a log		
12	Collect all necessary materials for the preparation		
13	Remove tear off caps		
14	Check collected materials in according to the preparation document	C	
15	Sign the preparation document for collecting and controlling the materials		
16	Collecting and controlling done by different operators	C	
17	Materials with a non-sterile surface: disinfection by wiping in accordance with disinfection procedure	C	
18	Wipes well impregnated		
19	Disinfected materials are placed in a sterile tray on a sterile surface (see [1], figure 1)		
20	Disinfect gloves regularly		
	**Set up by primary operator**		
21	Remove gloves and disinfect hands		
22	Put on sterile sleeves (outside LAF/SC)		
23	Put on sterile gloves without chance of outside contamination	C	
24	Check sterile gloves after putting on for tears or gaps		
	**Transfer materials into LAF/SC**		
25	Secondary operator: disinfect gloved hands, unwrap sterile pad partly in front of LAF/SC and present sterile site to primary operator		
26	Primary operator: place sterile pad on the right place inside LAF/SC		
27	Materials with a sterile surface<br>Secondary operator: unwrap partly in front of LAF/SC and present sterile site to primary operator	C	
28	No wrapped surface inside LAF/SC		
29	Primary operator: places SMD with open critical spots (open tubes, syringes and needles) on the sterile pad and full capped SMD as well as infusion bags outside the sterile pad (see [2], online supplementary figure 2)	C	

30	Secondary operator: present tray with disinfected materials to primary operator		
31	Primary operator: disinfected materials inside LAF/SC. Beware of:<br>• keeping first air on critical spots<br>• enough space to perform the preparation		
32	Primary and secondary operator work as a team		
	**Preparation (primary operator)**		
33	Open settle plate		
34	Enough distance between work location and LAF/SC front		
35	Unload the tray with disinfected materials; keep the tray inside LAF/SC for sampling waste during preparation		
36	Disinfect vial stoppers and ampoule necks (critical spots) in accordance with disinfection procedure by wiping; wait at least 30 sec before puncturing stopper	C	
37	Keep critical spots from sterile and non-sterile materials always in first air		
38	Execute aseptic manipulations in first air	C	
39	Enough distance between fingers and critical spots on materials	C	
40	Non-touch working technique	C	
41	Glove disinfection every 15 minutes	C	
42	Keep SMD with open critical spots on the sterile pad; no other materials on this pad	C	
43	Collect waste in empty tray		
44	Keep hands inside LAF/SC		
45	Keep LAF/SC well organized		
46	No social talks during preparation		
	**After preparation**		
47	Primary and secondary operator: transfer finished product outside LAF/SC		
48	Secondary operator: label product immediately after transfer	C	
49	Primary operator: Surface monitoring by contact plate (contact time at least 3 sec)		
50	Primary operator: glove print 5 fingers (contact time each finger at least 3 sec)		
51	Make LAF/SC empty and disinfect worktop by wiping		
52	Glove change or glove disinfection if a next preparation will follow		

References

1. Boom FA, Le Brun PPH, Boehringer S, Kosterink JGW, Touw DJ. Improving the aseptic transfer procedures in hospital pharmacies Part B: Disinfection methods for materials with a non-sterile surface. August 2019. Published online by Eu J Hosp Phar.
2. Boom FA, Le Brun PPH, Boehringer S, Kosterink JGW, Touw DJ. Improving the aseptic transfer procedures in hospital pharmacies Part C: Evaluation and redesign of the transfer process. October 2019. Published online by Eu J Hosp.
