# Supplementary material for: Reducing the risk of non-sterility of aseptic handling in hospital pharmacies, part B: risk control
Source: Eur J Hosp Pharm. 2020 May 8;28(6):325–30. doi: 10.1136/ejhpharm-2019-002179 (PMC8552189; doi:10.1136/ejhpharm-2019-002179)
Supplement: Supplementary data [file ejhpharm-2019-002179supp005.pdf]

SUPPLEMENTARY FILE 5

Risk assessment and risk control of the operator. D, detection; O, occurrence; RPN, risk prioritisation number; S, severity; SOP, standard operating procedure. F, G and H=sources of risk of non-sterility.

	sources of risk and risk reduction in 10 hospital pharmacies	remaining risk in 10 hospital pharmacies	S	O	D	RPN	additional risk reduction (1)	remaining risk	S	O	D	RPN
F	**Operators hands;** wearing sterile gloves, which are changed at least every hour; daily monitoring by glove print 5 fingers	glove damage	5	2	3	30	check gloves integrity immediately after putting them on and during processing	there is still a chance of glove damage	5	2	2	20
		surface contamination during putting on gloves	5	2	3	30	good putting on technique	there is still a chance of wrong putting on	5	2	2	20
		surface contamination during preparation	5	3	3	45	glove disinfection before start of each new preparation and in between every 15 min	disinfection forgotten	5	2	3	30
G	**Operators fore arm;** waring cleanroom clothing which is changed every day	surface contamination of the worktop	5	2	3	30	operator wears sterile sleeves which have to be changed after every session	unlikely	5	1	1	5
H	**Working procedure**; working with two operators; SOP; operators trained in aseptic techniques by broth simulations every year; process validation by broth simulation	deviation from SOPs	5	3	3	45	improving SOPs	not enough working discipline	5	2	3	30
		touching critical spots	5	4	4	80	additional training in non-touch working	there is still a substantial chance of touch	5	3	3	45
		a. crossflow: blocking first air at critical spots	5	2	3	30	operators are regularly audited	there is still a chance of blocking first air	5	1	2	10
		or b. downflow: blocking first air at critical spots	5	3	3	45	operators are regularly audited	there is still a chance of blocking first air	5	2	2	20

Supplementary file 5, continued

	additional risk reduction (2)	remaining risk	S	O	D	RPN	additional risk reduction (3)	cause	S	O	D	RPN
F	operators are regularly audited	unlikely	5	1	1	5	both operators correct each other	unlikely	5	1	1	5
	operators are regularly audited	unlikely	5	1	1	5	both operators correct each other	unlikely	5	1	1	5
	operators are regularly audited	there is still a chance of disinfection forgotten	5	1	2	10	both operators correct each other	unlikely	5	1	1	5
G												
H	operators are regularly audited	there is still a chance of deviation from SOPs	5	1	2	10	both operators correct each other	unlikely	5	1	1	5
	operators are regularly audited	there is still a chance of touch	5	2	2	20	both operators correct each other	still a chance of touch	5	1	2	10
	both operators correct each other	unlikely	5	1	1	5						
	both operators correct each other	still a chance of blocking first air	5	2	1	10						
