# Supplementary material for: Reducing the risk of non-sterility of aseptic handling in hospital pharmacies, part B: risk control
Source: Eur J Hosp Pharm. 2020 May 8;28(6):325–30. doi: 10.1136/ejhpharm-2019-002179 (PMC8552189; doi:10.1136/ejhpharm-2019-002179)
Supplement: Supplementary data [file ejhpharm-2019-002179supp004.pdf]

SUPPLEMENTARY FILE 4

Risk assessment and risk control of the transfer of materials. D, detection; LAF, laminar airflow cabinet; O, occurrence; RPN, risk prioritisation number; S, severity; SC, safety cabinet. D1, D2, E1 and E2=sources of risk of non-sterility.

	sources of risk and risk reduction in 10 hospital pharmacies	remaining risk in 10 hospital pharmacies	S	O	D	RPN	additional risk reduction (1)	remaining risk	S	O	D	RPN
D1	**Materials with a sterile surface** (sterile devices and infusion bags); unwrapping in front of LAF/SC	parts of outer layer inside LAF/SC	5	2	3	30	a logistic process to assure a low surface bio-burden of the outer layer [5]	no good aseptic transfer	5	1	3	15
D2	**Critical spots** (syringe tips, needles and the opening of tubes)	contact of critical spots with the work top of LAF/SC	5	4	3	60	putting down syringes, needles and open tubes on a sterile pad in LAF/SC [5]	no good use of sterile pad	5	2	2	20
E1	**Materials and equipment with a non-sterile surface** (ampoules, vials, bottles); disinfection by wiping with ethanol or isopropyl alcohol 70%	high surface bioburden before disinfection	5	3	3	45	a logistic process to assure a low surface bioburden [5]	deviation from the right logistic process	5	2	2	20
		disinfection improperly done	5	4	4	80	precisely described and validated disinfection procedure [4]	disinfected material and equipment is not monitored regularly	5	2	4	40
		recontamination of disinfected materials	5	3	3	45	measures to prevent recontamination [4,5]	recontamination still happens	5	2	3	30
E2	**Critical spots** (vial stoppers and ampoule necks); additional disinfection in LAF/SC by wiping with sterile ethanol or isopropyl alcohol 70%	additional disinfection improperly done	5	3	4	60	improved second disinfection technique	risk of no proper disinfection still exists	5	2	4	40

Supplementary file 4, continued

	additional risk reduction (2)	remaining risk	S	O	D	RPN	additional risk reduction (3) and (4)	remaining risk	S	O	D	RPN
D1	aseptic transfer is regularly audited	no good aseptic transfer still exists	5	1	2	10	both operators correct each other	unlikely	5	1	1	5
D2	use of sterile pad is regularly audited	unlikely	5	1	1	5	both operators correct each other	unlikely	5	1	1	5
E1	logistic process is regularly audited	deviation from the right logistic process still exists	5	1	2	10	both operators correct each other	unlikely	5	1	1	5
	regular monitoring of disinfected materials and equipment [4]	risk of no proper disinfection still exists	5	2	2	20	(3) disinfection is regularly audited and (4) both operators correct each other	unlikely	5	1	1	5
	measures are regularly audited	recontamination still happens	5	1	2	10	both operators correct each other	unlikely	5	1	1	5
E2	disinfection procedure is regularly audited	no assurance of a sterile surface	5	1	3	15	both operators correct each other	still no assurance of a sterile surface	5	1	2	10
